# Pancreatic cancer in the era of precision medicine: challenges, advances, and the future of therapeutic strategies

**DOI:** 10.1186/s43046-025-00323-w

**Published:** 2025-10-06

**Authors:** Heslley Machado Silva, Reginaldo Cruz Alves Rosa

**Affiliations:** 1 State University of Minas Gerais, ibirité, Brazil; 2 University Center of Formiga, Formiga, Brazil; 3 California Institute of Technology, Pasadena, United States; 4 University of Southern California, Los Angeles, United States

**Keywords:** Pancreatic cancer, Immunotherapy, Late-stage diagnosis, Personalized medicine, Targeted therapies

## Abstract

**Background:**

Pancreatic cancer stands among the most aggressive and fatal malignancies, with a steadily increasing incidence worldwide. Its clinical significance lies not only in its high mortality rate but also in the challenges associated with its early detection, limited therapeutic efficacy, and substantial impact on healthcare systems. The asymptomatic nature of the disease in its initial stages, combined with the absence of reliable early biomarkers, contributes to frequent late-stage diagnoses, significantly compromising treatment success and patient survival. Furthermore, the biological complexity of pancreatic tumors – often marked by specific genetic mutations and a highly immunosuppressive tumor microenvironment – drives resistance to conventional therapies, exacerbating clinical management difficulties.

**Main body:**

This narrative review offers a comprehensive synthesis of the major therapeutic challenges and recent advances in pancreatic cancer management over the past fifteen years. The main challenges include delayed diagnosis, the presence of treatment-resistant tumor subtypes, and the considerable financial burden of care. Particular attention is given to the tumor microenvironment, which impedes drug delivery and immune system activation due to its dense fibrotic stroma and immunosuppressive cellular composition. The review also explores emerging therapeutic strategies, including combination chemotherapy regimens such as folinic acid, fluorouracil, irinotecan, and oxaliplatin, and advanced radiotherapy techniques that aim to enhance precision while minimizing tissue damage. Furthermore, novel immunotherapeutic approaches – including messenger RNA-based vaccines and engineered cellular therapies – show promising results in stimulating targeted immune responses, although they face substantial barriers due to the tumor’s immune evasion mechanisms. Targeted therapies focused on specific genetic alterations, especially those involving KRAS mutations, are also highlighted as potential breakthroughs. The review concludes by emphasizing the relevance of personalized medicine, with biomarker-driven strategies and three-dimensional tumor models offering more tailored and potentially effective interventions.

**Conclusion:**

Despite meaningful progress in the development of innovative therapeutic modalities, pancreatic cancer continues to present profound medical and scientific challenges. Integrating personalized, interdisciplinary approaches and advancing early diagnostic tools remain essential steps toward improving clinical outcomes and extending survival in affected patients. This review underscores the urgent need for continued research to transform current insights into effective and accessible treatment strategies.

## Introduction

Pancreatic cancer is one of the most aggressive and lethal malignancies, holding a prominent position in oncological discussions due to its high mortality rate and the complexity associated with its diagnosis and treatment [1]. The significance of this type of cancer lies not only in its increasing incidence across various countries but also in its devastating impact on patients' quality of life and the burden it places on healthcare systems [2, 3].

The global incidence of pancreatic cancer has been consistently increasing over the years, particularly in developed countries, although the underlying reasons for this trend remain poorly understood [4]. Potential risk factors include population aging, obesity, increased alcohol consumption, smoking, and type 2 diabetes. However, the intricate interplay between genetic predisposition, lifestyle, and environmental influences still requires further investigation [2, 5, 6]. Smoking accounts for approximately 25% of pancreatic cancer cases globally, while obesity and type 2 diabetes increase the risk by 20–50%, depending on duration and severity of metabolic alterations [7]. This lack of a comprehensive understanding hampers preventive interventions, making it more challenging to implement effective disease control strategies [8, 9].

Pancreatic cancer presents with a highly aggressive progression, which is partially explained by a high propensity for early metastasis, a highly immunosuppressive tumor microenvironment, and intrinsic resistance to existing therapies [10, 11]. However, the exact mechanisms underlying this resistance and aggressiveness remain active research areas, emphasizing the complexity of developing more effective therapeutic approaches [12].

Pancreatic ductal adenocarcinoma (PDAC), the most common form of pancreatic cancer, exhibits distinct molecular subtypes, such as Classical and Basal-like, which demonstrate varied responses to treatment and clinical outcomes. Classical subtypes are generally more responsive to chemotherapy, while Basal-like tumors are typically more aggressive, have higher stromal content, and exhibit resistance to standard therapies. Understanding this biological heterogeneity is critical to the advancement of precision-based and personalized therapeutic strategies aimed at improving clinical outcomes.

Furthermore, the costs associated with pancreatic cancer treatment are substantial, reflecting the need for multidisciplinary interventions and the intensive use of advanced medical technologies [13]. Disease management involves sophisticated imaging techniques, complex surgical procedures, and multimodal therapies, with estimated treatment costs reaching tens of thousands of dollars per patient, depending on the disease stage and healthcare system [14]. This financial burden poses significant challenges to healthcare infrastructures, particularly in middle- and low-income countries [15]. In the United States, the average cost per pancreatic cancer patient exceeds USD 100,000, while in Brazil and India, access to standard chemotherapy remains inconsistent due to infrastructure and budgetary constraints [13, 16].

Late-stage diagnosis remains one of the major obstacles to successful disease management, as most patients are diagnosed at advanced stages when surgical resection is no longer viable [17, 18]. This challenge arises due to the absence of specific symptoms in the early stages and the lack of reliable biomarkers for early detection [19, 20].

The limited post-diagnosis life expectancy of pancreatic cancer is one of the key factors that make this malignancy particularly challenging. It is estimated that the global five-year survival rate remains below 10%, even in countries with advanced healthcare systems, primarily due to the delayed detection and the biologically aggressive nature of the tumor [8, 17]. The median survival for most patients ranges from only 6 to 12 months after diagnosis, particularly in advanced stages when curative treatment is not available [21, 22]. This grim outlook reflects not only the difficulty in early detection but also the limitations of currently available therapeutic approaches, which have yet to halt tumor progression and metastasis effectively [2]. Thus, the poor survival expectancy underscores the urgent need for more effective strategies, both in early diagnosis and in the development of innovative therapies that could improve patient prognosis [11, 12].

Although advances in modern therapies, such as combination chemotherapy and radiotherapy, have resulted in modest improvements in survival, the overall benefits remain limited. Radiotherapy, for instance, has been employed both as a standalone treatment and in combination with chemotherapeutic agents such as gemcitabine. However, its impact on survival is generally modest due to the radiation-induced resistance of pancreatic tumors [23]. Additionally, stereotactic body radiotherapy (SBRT) has shown promise in reducing tumor volumes, but its application remains restricted to specialized centers [24]. Other approaches, including targeted therapies and immunotherapies, also face significant challenges, mainly due to the molecular heterogeneity of pancreatic tumors and the lack of effective predictive biomarkers [25, 26].

Additionally, emerging early detection strategies, including liquid biopsy and metabolomic profiling, offer new avenues for improving the prognosis of pancreatic cancer patients by enabling earlier intervention. Early detection programs, such as circulating tumor DNA (ctDNA) analysis and multi-omic diagnostic platforms, are under active investigation and may play a crucial role in changing the clinical landscape of this malignancy. Thus, understanding pancreatic cancer's multifaceted difficulties remains a prerequisite to drive innovation in treatment paradigms.

This review article aims to provide a comprehensive synthesis of the major challenges in treating pancreatic cancer while discussing the most promising therapeutic advancements developed over the past 15 years, with a particular focus on innovative approaches that seek to improve diagnostic and treatment prospects for this devastating disease.

In recent years, precision medicine has emerged as a transformative approach in oncology. Unlike conventional therapies that adopt a one-size-fits-all strategy, precision medicine tailors’ treatment based on individual molecular, genetic, and environmental profiles. This approach enables more effective interventions, reduces unnecessary toxicity, and improves clinical outcomes by aligning therapy selection with predictive biomarkers and specific tumor characteristics [27, 28].

## Methodology

This article adopts a narrative review as its methodological approach, considering the necessity of comprehensively and critically exploring the current state of knowledge on pancreatic cancer. A narrative review is particularly suited for integrating multiple aspects of a complex topic, such as the therapeutic challenges and advancements in the treatment of pancreatic cancer, allowing for a detailed and contextually enriched analysis of the available information [29, 30].

The research was conducted using academically recognized databases, including PubMed, Scopus, Web of Science, and Google Scholar, covering the period from 2008 to 2024. This timeframe was chosen to encompass significant advancements in the field over the past 15 years. The search strategy employed strategic keyword combinations, applied with Boolean operators to ensure both precision and comprehensiveness. The selected terms included: "pancreatic cancer," "treatment challenges," "therapeutic advances," "survival," "chemotherapy," "radiotherapy," "immunotherapy," and "targeted therapy."

The selection process for studies followed a rigorous three-step procedure:

1. Title Screening: The initial triage was conducted based on titles, aiming to identify publications potentially relevant to the objectives of this review. Studies that clearly did not align with the research focus were excluded at this step.
2. Abstract Review: In the second phase, abstracts were carefully analyzed to assess their adherence to the inclusion criteria and ensure alignment with the thematic axes of the study. Priority was given to articles containing relevant and up-to-date information on pancreatic cancer treatment challenges and therapeutic advancements.
3. Full-Text Analysis: Studies that met the criteria in the previous steps underwent a comprehensive reading, with a focus on detailed data extraction. This analysis aimed to highlight key findings related to the obstacles in treating pancreatic cancer and the most recent advances in therapeutic strategies.

The inclusion criteria for study selection encompassed the following parameters: (1) Peer-reviewed articles to ensure methodological rigor and scientific reliability. (2) Publications in English or Portuguese, allowing for a broad and diverse dataset while maintaining accessibility. (3) Studies focused on major therapeutic challenges or advancements in pancreatic cancer treatment, ensuring relevance to the research objectives. (4) Data published within the 2008–2024 period to capture recent developments and contextualize findings within contemporary scientific progress.

Exclusion criteria included duplicate articles, studies lacking direct relevance to the topic, and publications that did not meet the outlined parameters were excluded.

**Fig. 1:**
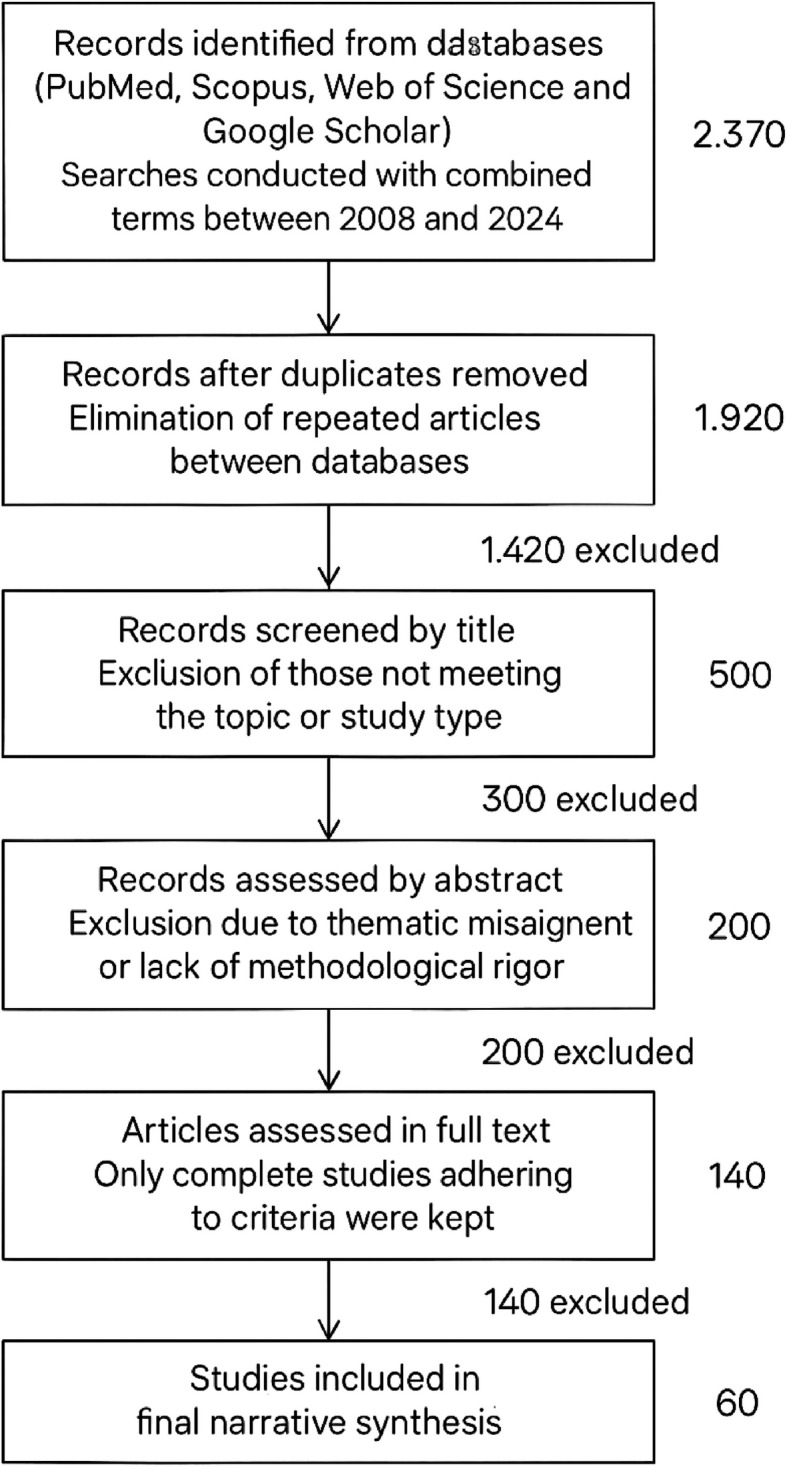
PRISMA diagram

A flow diagram based on the PRISMA guidelines was developed to enhance methodological transparency and illustrate the article selection process (Fig. 1).

### Thematic Axes

The review was structured around two main thematic axes: (1) Key challenges in pancreatic cancer treatment, including late diagnosis, resistance to conventional therapies, and associated treatment costs. (2) Major therapeutic advancements, encompassing combination chemotherapy, novel radiotherapy approaches, immunotherapies, and targeted therapies.

This methodological approach aimed to synthesize the most relevant and up-to-date data available in literature, providing a comprehensive understanding of the challenges and opportunities in managing this highly lethal malignancy. The integration of results establishes a solid foundation for future investigations and innovative treatment strategies, contributing to improved clinical outcomes for pancreatic cancer patients.

The majority of the included studies comprised peer-reviewed original research articles, clinical trials, and systematic reviews. Priority was given to studies with high methodological rigor, large sample sizes, and multicenter collaboration. Clinical relevance and data consistency were also considered in the evaluation of study quality.

## Results

The literature review on pancreatic cancer revealed significant insights across two major thematic axes: challenges in treatment and therapeutic advancements.

### Challenges in pancreatic cancer treatment

The absence of specific symptoms in the early stages of pancreatic cancer often results in late-stage diagnoses, significantly compromising the effectiveness of therapeutic interventions [7, 17, 31]. Additional studies confirm this observation, emphasizing that late detection is strongly associated with high mortality rates due to the disease's silent progression [32–34]. Moreover, the lack of reliable early biomarkers and non-specific clinical manifestations contribute to diagnostic delays, making early screening strategies an urgent necessity [17, 35].

The intrinsic biology of pancreatic cancer, particularly its association with genetic mutations, notably in the *KRAS* gene, contributes to its highly aggressive behavior and resistance to conventional treatments [36, 37]. Recent studies indicate that specific *KRAS* mutations, such as G12D, G12V, and G12R, are found in over 90% of pancreatic tumors, positioning them as critical targets for novel therapies [38–40]. Moreover, the presence of *KRAS* mutations activates signaling pathways that enhance cell survival and proliferation, thereby driving resistance to chemotherapy and radiotherapy [41]. The emergence of KRAS-specific inhibitors and their limited efficacy due to resistance mechanisms further underscore the complexity of treating pancreatic ductal adenocarcinoma (PDAC) and highlight the need for combination therapeutic approaches [42].

In addition to KRAS, mutations in TP53, CDKN2A, and SMAD4 are frequently observed in pancreatic ductal adenocarcinoma (PDAC). TP53 mutations, present in ~ 70% of cases, contribute to genomic instability and resistance to apoptosis. CDKN2A mutations disrupt cell cycle control, while SMAD4 inactivation is associated with poor prognosis and metastatic potential [43]. Together, these alterations reflect the complex genetic landscape that shapes tumor behavior and therapeutic response.

The dense stroma surrounding pancreatic tumor cells creates physical and biological barriers that hinder the penetration and efficacy of therapeutic agents [44, 45]. The PDAC tumor microenvironment is characterized by an abundant extracellular matrix and cancer-associated fibroblasts (CAFs), which promote tumor progression and therapeutic resistance [46–48]. Additionally, the rigidity and anisotropy of the desmoplastic matrix facilitate tumor invasion, mediated by specific signaling pathways [49].

The immunosuppressive nature of the pancreatic tumor microenvironment is infiltrated by immunosuppressive cells, including M2-type macrophages, regulatory T cells (Tregs), and neutrophils, which impair the recruitment and anti-tumor activity of cytotoxic immune cells, such as CD8 + T lymphocytes and natural killer (NK) cells [50, 51], posing significant challenges to treatment. Strategies aimed at modulating the tumor stroma, including the use of plant-derived compounds, have been proposed to enhance chemotherapy and immunotherapy efficacy [52].

The genetic and adaptive heterogeneity of pancreatic tumors leads to resistance to chemotherapy and radiotherapy, significantly limiting effective treatment options [53, 54]. Studies suggest that the intratumoral heterogeneity in PDAC is driven by cooperative cellular subpopulations, contributing to therapy resistance [46, 55, 56]. Moreover, cellular plasticity in PDAC is linked to the persistence of cancer stem cell-like states, further sustaining resistance to conventional therapies [57].

Additionally, epigenetic alterations play a critical role in modulating gene expression, enabling tumor cells to adapt and survive under therapeutic stress [58]. The identification of specific molecular subtypes in PDAC has provided valuable insights into variability in treatment response, reinforcing the necessity for personalized therapeutic approaches [52].

Given these formidable barriers, the past decade has seen an acceleration of research focused on novel therapeutic strategies that aim to counteract pancreatic cancer’s inherent resistance.

### Advancements in treatment

**T**he FOLFIRINOX (folinic acid, 5-fluorouracil, irinotecan, and oxaliplatin) regimen has significantly improved overall survival for patients with pancreatic cancer. However, it is associated with substantial toxicity, requiring careful management [59, 60]. Recent studies indicate that patients treated with FOLFIRINOX have a median overall survival of 11.1 months, compared to 6.8 months for gemcitabine monotherapy, though it also presents a higher incidence of severe adverse events [59–61]. Additionally, the combination of gemcitabine with nab-paclitaxel has shown a median overall survival of 8.5 months, offering an alternative with a different toxicity profile [62]. While FOLFIRINOX remains one of the most effective chemotherapy regimens for metastatic pancreatic cancer, its use is limited by significant hematologic and gastrointestinal toxicities [63, 64]. Median overall survival was 11.1 months for FOLFIRINOX versus 6.8 months for gemcitabine monotherapy (Table 1)[59]. These findings underscore the need to balance efficacy and tolerability when selecting chemotherapy regimens for pancreatic cancer patients.

**Table 1:** Characteristics of the main chemotherapy regimens for pancreatic cancer

Regimen	Median OS (months)	Main Toxicities	Clinical Indication
FOLFIRINOX	11.1	Neutropenia, diarrhea, fatigue	Fit patients with metastatic disease
Gemcitabine + nabpaclitaxel (GA)	8.5	Neuropathy	Alternative for less fit patients
Gemcitabine (monotherapy)	6.8	Mild hematologic	Patients with poor performance status

The development of mutation-specific inhibitors, particularly for the *KRAS* G12C mutation, offers new therapeutic perspectives, though these approaches are still in the early stages of research [65, 66]. Recent studies indicate that sotorasib, a *KRAS* G12C inhibitor, demonstrated an objective response rate of 21% in patients diagnosed with advanced pancreatic cancer, although the median duration of response was limited [67–70]. Additionally, combining KRAS inhibitors with immunotherapies has been explored as a potential strategy to enhance therapeutic efficacy in preclinical pancreatic cancer models [69, 71]. However, *KRAS* mutational heterogeneity presents significant challenges, as the predominantly occurring G12D mutation currently lacks widely tested and clinically approved inhibitors [72]. In mismatch repair-deficient (dMMR) tumors, immune checkpoint inhibitors such as pembrolizumab have shown clinical efficacy, highlighting the importance of biomarker-driven therapy selection [73]. These advancements underscore the critical need for ongoing research to evaluate the efficacy and safety of targeted therapies in pancreatic cancer patients.

Therapeutic approaches aimed at stimulating the immune system, including cancer vaccines and cellular therapies, are currently under investigation, with promising preliminary results in clinical trials [74, 75]. Recently, an mRNA-based vaccine for pancreatic cancer developed by BioNTech and Genentech demonstrated in a phase 1 clinical trial that half of the participants developed tumor-specific T cells after treatment, indicating a significant adaptive immune response [76]. Additionally, a recent study has identified tertiary lymphoid structures (TLSs) within pancreatic tumors, which may serve as potential targets for immunotherapy, paving the way for more effective treatments [77, 78]. Cell-based therapies comprising the infusion of genetically engineered immune systems are also being explored in pancreatic cancer. For instance, T cells engineered with a T cell receptor designed to recognize pancreatic tumors harboring the *KRAS* G12D mutation were able to mediate the regression of visceral metastases in a 67-year-old patient diagnosed with pancreatic cancer [79]. Furthermore, chimeric antigen receptor (CAR) T cell therapy has also shown encouraging results in pancreatic cancer patients [80, 81]. These advancements suggest that immunotherapy – particularly personalized vaccines and cellular therapies – may become a viable option for combating pancreatic cancer. Nevertheless, CAR T therapies face limitations in pancreatic cancer due to antigenic heterogeneity, immune escape, and the immunosuppressive tumor microenvironment. However, further research is necessary to confirm their long-term efficacy and safety.

Innovative radiotherapy techniques, such as irreversible electroporation (IRE) and stereotactic body radiotherapy (SBRT), enable the precise administration of high radiation doses while minimizing damage to adjacent tissues [82, 83]. Recent studies indicate that IRE is a non-thermal ablation technique that utilizes electrical pulses to induce tumor cell apoptosis, while preserving critical structures such as blood vessels and bile ducts. This makes IRE particularly useful for patients with locally advanced pancreatic tumors, where conventional surgical resection is not feasible [84–86]. Additionally, SBRT offers the advantage of delivering ablative radiation doses in a limited number of sessions with high precision, leading to effective local tumor control while maintaining an acceptable toxicity profile [87]. The combination of these advanced radiotherapy techniques with systemic therapies is being actively explored to enhance oncological outcomes. However, further studies are required to establish optimized treatment protocols and assess long-term benefits in pancreatic cancer management [88]. SBRT is primarily indicated for patients with localized, non-resectable pancreatic tumors who cannot undergo surgery. Its main limitation is proximity to radiosensitive organs, requiring careful planning to avoid gastrointestinal toxicity. IRE is indicated for locally advanced tumors near vital structures such as blood vessels and bile ducts, where thermal ablation would be unsafe. However, it is limited by the need for general anesthesia and availability of specialized equipment [89].

The analysis of tumor biomarkers enables the selection of more effective therapies for specific patient subgroups, thereby enhancing treatment efficacy [90, 91]. Recent studies indicate that the identification of germline genetic mutations in pancreatic cancer can aid in detecting individuals with a higher predisposition to the disease and inform individualized treatment decisions. Patients with BRCA1/2 mutations respond better to platinum-based chemotherapy and may benefit from PARP inhibitors like olaparib, which demonstrated progression-free survival benefit in phase III trials [92]. Biomarkers such as BRCA1/2 and dMMR enable the selection of patients for targeted therapies, such as PARP inhibitors or immune checkpoint inhibitors. Functional 3D organoids have been developed to simulate tumor response in vitro, offering predictive insights for treatment planning. This approach holds potential benefits for early diagnosis and improved patient survival [93–95]. Additionally, the application of three-dimensional (3D) pancreatic cancer models, integrated with optical sensors, allows for non-invasive tumor metabolism monitoring and drug screening, contributing to the development of tailored therapeutic approaches [96–98]. These advancements highlight the critical role of personalized medicine in optimizing pancreatic cancer treatment. However, further research is needed to validate the clinical efficacy of these strategies and integrate them into routine oncological practice.

Recent technological advances have significantly enhanced the prospects for early detection of pancreatic cancer—an essential factor in improving patient prognosis. Among the most promising innovations are liquid biopsy techniques, which allow for the non-invasive identification of circulating tumor DNA (ctDNA), and metabolomic profiling, which detects alterations in metabolic signatures associated with early-stage tumor development. A recent pilot study employing a multi-omic diagnostic platform demonstrated a sensitivity of 83% for early-stage pancreatic tumors, highlighting the potential of integrated biomarker approaches to overcome the current limitations of traditional diagnostic tools [99]. These findings underscore the importance of continued investment in molecular screening strategies, which may enable earlier clinical intervention, improve resectability rates, and ultimately enhance survival outcomes in pancreatic cancer.

## Discussion

Pancreatic cancer remains one of the most challenging malignancies today, characterized by biological complexity, high tumor aggressiveness, and significant clinical and structural obstacles such as late-stage diagnosis and limited therapeutic efficacy. An in-depth analysis of these challenges and emerging therapeutic advancements underscores the urgent need for integrated and innovative strategies to improve clinical outcomes and enhance patients' quality of life.

One of the greatest obstacles to the treatment of pancreatic cancer is late diagnosis. The absence of specific symptoms in the early stages of the disease often leads to detection at advanced stages, when surgical resection of tumors is no longer viable and treatment options are limited [7, 17, 31]. This silent progression is further aggravated by the disease's intrinsic biological aggressiveness, often driven by mutations in genes such as *KRAS* and *TP53* [36, 40]. In addition to modulating cellular signaling pathways related to tumor development and progression, these mutations are also implicated in mechanisms of therapeutic resistance, representing one of the main challenges in clinical management [39, 42].

In addition to tumor biology and delayed detection, the surrounding tumor microenvironment and acquired resistance mechanisms present substantial therapeutic barriers. The tumor microenvironment also plays a crucial role in the difficulty of treating this malignancy. In pancreatic cancer, the stroma accounts for approximately 50% to 80% of the total tumor volume and is characterized by the presence of desmoplasia, composed of fibroblasts and excessive deposition of extracellular matrix components, which restricts the penetration of therapeutic agents into the tumor microenvironment and limits their antitumor activity [100]. Consequently, several preclinical studies and clinical trials investigating the therapeutic potential of modulators targeting various components of the pancreatic cancer stroma have been initiated.

In addition to the presence of a dense stroma, the tumor microenvironment in pancreatic cancer is highly immunosuppressive, with a reduced presence of natural killer (NK) cells and CD8 + T lymphocytes, both of which play a fundamental role in the antitumor immune response [101]. The limited infiltration of cytotoxic lymphocytes is further exacerbated by the abundant presence of macrophages, neutrophils, regulatory T cells (Tregs), and other immunosuppressive immune cells. These cells promote T cell exhaustion and immune exclusion through the expression of cytokines, chemokines, and other extracellular components within the pancreatic tumor microenvironment [102, 103]. Thus, innovative therapeutic strategies aimed at depleting immunosuppressive cells infiltrating the pancreatic tumor microenvironment and enhancing the infiltration of cytotoxic immune cells are necessary to overcome the hostile tumor microenvironment that currently limits the effectiveness of standard pancreatic cancer therapies.

Molecular subtypes of PDAC, particularly Classical and Basal-like, have distinct biological and clinical behaviors. Classical subtypes tend to respond better to chemotherapy, whereas Basal-like tumors exhibit higher aggressiveness, increased stromal content, and intrinsic resistance to standard treatments [104]. These differences underscore the need for subtype-specific therapeutic strategies.

The tumor microenvironment plays a central role in treatment resistance in pancreatic cancer. Rather than serving as a passive background, the stromal and immune components actively shape tumor progression and therapeutic failure. Given the dense desmoplastic matrix and prevalence of immunosuppressive cells, emerging strategies now focus on targeting stromal remodeling and reprogramming immune cell populations to enhance treatment response. Future research must aim to define predictive biomarkers within the TME and identify optimal therapeutic combinations to overcome this biological barrier.

Nonetheless, recent therapeutic developments offer encouraging perspectives and reshaping treatment paradigms in pancreatic cancer. Combination chemotherapy regimens, notably FOLFIRINOX, have demonstrated significant improvements in overall survival for patients with advanced pancreatic cancer. However, their clinical benefit is frequently counterbalanced by substantial hematologic and gastrointestinal toxicities, which may limit their use, particularly in elderly or frail individuals[59, 61].. As an alternative, the combination of gemcitabine and nab-paclitaxel offers a more tolerable toxicity profile, though its survival benefit remains comparatively modest[62]..

In parallel, targeted therapies – particularly those aimed at *KRAS* mutations—have gained attention as potential breakthroughs. The development of *KRAS* G12C inhibitors, such as sotorasib, has shown encouraging results in a subset of patients; however, therapeutic resistance, limited response durability, and the predominance of other *KRAS* variants (e.g., G12D) present considerable challenges. Moreover, the molecular heterogeneity of pancreatic tumors and the lack of robust predictive biomarkers continue to hinder broader applicability [65, 70]. These limitations underscore the need for combinatorial strategies and biomarker-driven approaches to maximize the clinical potential of both cytotoxic and molecularly targeted therapies in pancreatic cancer.

Immunotherapy has emerged as a promising frontier in the treatment of pancreatic cancer, particularly through the development of personalized mRNA vaccines. Early-phase clinical trials have demonstrated the capacity of these vaccines to elicit robust and tumor-specific immune responses, offering new hope in a historically treatment-refractory malignancy. Additionally, cell-based strategies, including adoptive T cell therapies such as TCR-engineered lymphocytes and chimeric antigen receptor (CAR) T cells, have shown potential in overcoming antigen-specific immune evasion in selected patients.

Nonetheless, the highly immunosuppressive tumor microenvironment (TME) of PDAC continues to pose a formidable obstacle to the efficacy of these therapies. The abundance of regulatory T cells, tumor-associated macrophages, and myeloid-derived suppressor cells, along with dense stromal barriers, collectively impairs the infiltration and activity of cytotoxic immune effectors. Overcoming this immunologic exclusion remains a critical challenge [74, 76, 105]. Current evidence suggests that combination strategies, integrating immunotherapies with chemotherapy, radiotherapy, or stromal modulators, may enhance therapeutic penetration and immune activation. Further clinical validation is necessary to define optimal protocols, identify predictive biomarkers of response, and ensure the safe and durable application of immunotherapeutic approaches in pancreatic cancer.

Predictive biomarkers such as tumor mutational burden (TMB), human leukocyte antigen (HLA) diversity, and the presence of tertiary lymphoid structures (TLSs) are under investigation for their roles in modulating immunotherapy response. TLSs have been associated with favorable prognosis and improved T cell recruitment, while elevated TMB and specific HLA genotypes may correlate with increased neoantigen presentation and immune activation [106, 107].

Complementing these strategies, advanced radiotherapy, including techniques such as irreversible electroporation and stereotactic body radiotherapy (SBRT), offers greater precision and local disease control, although its integration with systemic therapies still requires further studies [83, 85].

Cell-based therapies, such as CAR T cells and TCR T cells, represent a promising therapeutic approach, with remarkable results in hematologic malignancies, such as leukemias and lymphomas [108]. However, the highly immunosuppressive tumor microenvironment present in most solid tumors, including pancreatic cancer, is undoubtedly one of the major factors limiting the clinical response of pancreatic cancer and other solid tumors treated with CAR T cells, TCR T cells, and other cellular therapies [109]. Thus, several studies have indicated that cell-based therapies alone may not be sufficient to achieve satisfactory clinical responses, supporting the use of these therapies in combination with other therapeutic agents, such as chemotherapy [110], radiotherapy [111], and other immunotherapy modalities [112–114].

The paradigm of personalized medicine has become increasingly central to the clinical management of pancreatic cancer, offering the potential to tailor treatments based on the molecular and genetic profile of each patient. The identification of actionable biomarkers – such as mutations, mismatch repair deficiency (dMMR), and other germline or somatic alterations – enables stratified therapeutic strategies, including the use of PARP inhibitors and immune checkpoint blockade, which have demonstrated efficacy in biomarker-selected populations.

Moreover, the application of three-dimensional (3D) tumor models and patient-derived organoids has opened new avenues for ex vivo testing of drug sensitivity, allowing for the simulation of therapeutic responses prior to clinical administration. These functional precision oncology platforms offer a means to optimize regimen selection, reduce trial-and-error prescribing, and accelerate the development of individualized therapeutic protocols[96, 97].

However, the clinical translation of these strategies remains in its early stages, with challenges including standardization of testing platforms, integration into routine care pathways, and cost-effectiveness evaluations. Furthermore, the therapeutic intensity of many current regimens necessitates careful consideration of functional outcomes and quality of life, particularly in older or frail individuals. Adverse effects such as fatigue, peripheral neuropathy, and gastrointestinal toxicity may compromise daily functioning and long-term well-being, highlighting the importance of shared decision-making, comprehensive geriatric assessment, and supportive care integration throughout the treatment continuum.

Looking forward, future directions in personalized oncology will require the convergence of multi-omic profiling, real-time liquid biopsy monitoring, and AI-driven decision tools to deliver truly dynamic, patient-centered cancer care. As these technologies evolve, robust clinical validation and equitable access will be critical to ensuring that precision medicine fulfills its promise in the context of pancreatic cancer [115].

Two critical research priorities emerge for the near future. First, the integration of multi-omic profiling – including genomics, transcriptomics, and metabolomics – should be expanded to refine patient stratification and identify novel therapeutic targets. Second, the establishment of prospective screening cohorts for high-risk populations (e.g., BRCA mutation carriers or patients with new-onset diabetes) may enable early diagnosis and improve surgical resection rates.

## Conclusion

The current landscape of pancreatic cancer is defined by significant challenges, ranging from late diagnosis and the tumor's aggressive biology to the barriers imposed by the tumor microenvironment and therapeutic resistance. However, advances in chemotherapy, targeted therapies, immunotherapy, advanced radiotherapy, and personalized medicine offer promising perspectives. Further research integrating interdisciplinary and personalized approaches is essential to translate these advancements into tangible clinical benefits. These findings reaffirm the necessity of integrated, biomarker-driven, and patient-centered strategies to overcome the multifactorial complexity of pancreatic cancer.

## Data Availability

No datasets were generated or analysed during the current study.

## References

[1] Siegel RL, Miller KD, Wagle NS, Jemal A. Cancer statistics, 2023. CA Cancer J Clin. 2023;73:17–48. 36633525 10.3322/caac.21763

[2] Rawla P, Sunkara T, Gaduputi V. Epidemiology of pancreatic cancer: global trends, etiology and risk factors. World J Oncol. 2019;10:10. 30834048 10.14740/wjon1166 PMC6396775

[3] Yu Z, et al. Differences in the incidence and mortality of digestive cancer between Global Cancer Observatory 2020 and Global Burden of Disease 2019. Int J Cancer. 2024;154:615–25. 37750191 10.1002/ijc.34740

[4] McGuigan C, Reynolds R, Wiedmer D. Poverty and climate change: assessing impacts in developing countries and the initiatives of the international community. London Sch Econ Consult. Proj Overseas Dev Inst. 2002;1–40. https://odi.org/documents/2557/3449.pdf.

[5] Ilic M, Ilic I. Epidemiology of pancreatic cancer. World J Gastroenterol. 2016;22:9694. 27956793 10.3748/wjg.v22.i44.9694 PMC5124974

[6] Luo W, et al. Epidemiology of pancreatic cancer: New version, new vision. Chin J Cancer Res. 2023;35:438. 37969957 10.21147/j.issn.1000-9604.2023.05.03 PMC10643340

[7] Garrido-Laguna I, Hidalgo M. Pancreatic cancer: from state-of-the-art treatments to promising novel therapies. Nat Rev Clin Oncol. 2015;12:319–34. 25824606 10.1038/nrclinonc.2015.53

[8] Bray F, et al. Global cancer statistics 2022: GLOBOCAN estimates of incidence and mortality worldwide for 36 cancers in 185 countries. CA Cancer J Clin. 2024;74:229–63. 38572751 10.3322/caac.21834

[9] Halbrook CJ, Lyssiotis CA, di Magliano MP, Maitra A. Pancreatic cancer: advances and challenges. Cell. 2023;186:1729–54. 37059070 10.1016/j.cell.2023.02.014 PMC10182830

[10] Pavlova NN, Thompson CB. The emerging hallmarks of cancer metabolism. Cell Metab. 2016;23:27–47. 26771115 10.1016/j.cmet.2015.12.006 PMC4715268

[11] Hidalgo M, et al. Addressing the challenges of pancreatic cancer: future directions for improving outcomes. Pancreatology. 2015;15:8–18. 25547205 10.1016/j.pan.2014.10.001

[12] Ryan DP, Hong TS, Bardeesy N. Pancreatic adenocarcinoma. N Engl J Med. 2014;371:1039–49. 25207767 10.1056/NEJMra1404198

[13] Cipora E, et al. Treatment costs and social burden of pancreatic cancer. Cancers (Basel). 2023;15:1911. 36980796 10.3390/cancers15061911 PMC10047484

[14] Kaye DR, et al. Costs of cancer care across the disease continuum. Oncologist. 2018;23:798–805. 29567821 10.1634/theoncologist.2017-0481 PMC6058326

[15] Khan MA. et al. Morbidity and mortality following surgery for pancreatic cancer in low‐and middle‐income countries: a systematic review and meta‐analysis. J Surg Oncol. 2024. 10.1002/jso.27946. 10.1002/jso.27946 PMC12014855 39444276

[16] Arjani S, et al. Neoadjuvant treatment versus upfront surgery in resectable pancreatic cancer: a cost-effectiveness analysis. JCO Oncol Pract. 2023;19:e439–48. 36548928 10.1200/OP.22.00536

[17] McGuigan A, et al. Pancreatic cancer: a review of clinical diagnosis, epidemiology, treatment and outcomes. World J Gastroenterol. 2018;24:4846. 30487695 10.3748/wjg.v24.i43.4846 PMC6250924

[18] Singhi AD, Koay EJ, Chari ST, Maitra A. Early detection of pancreatic cancer: opportunities and challenges. Gastroenterology. 2019;156:2024–40. 30721664 10.1053/j.gastro.2019.01.259 PMC6486851

[19] Kenner BJ, et al. Early detection of pancreatic cancer—a defined future using lessons from other cancers: a white paper. Pancreas. 2016;45:1073–9. 27518362 10.1097/MPA.0000000000000701 PMC4993121

[20] Shah Y, et al. Advancements in early detection and screening strategies for pancreatic cancer: from genetic susceptibility to novel biomarkers. J Clin Med. 2024;13:4706. 39200847 10.3390/jcm13164706 PMC11355237

[21] Rahib L, et al. Projecting cancer incidence and deaths to 2030: the unexpected burden of thyroid, liver, and pancreas cancers in the United States. Cancer Res. 2014;74:2913–21. 24840647 10.1158/0008-5472.CAN-14-0155

[22] He R, Jiang W, Wang C, Li X, Zhou W. Global burden of pancreatic cancer attributable to metabolic risks from 1990 to 2019, with projections of mortality to 2030. BMC Public Health. 2024;24:456. 38350909 10.1186/s12889-024-17875-6 PMC10865635

[23] Myrehaug S, et al. Stereotactic body radiotherapy for pancreatic cancer: recent progress and future directions. Expert Rev Anticancer Ther. 2016;16:523–30. 26999329 10.1586/14737140.2016.1168698

[24] Mahadevan A, et al. Stereotactic body radiotherapy and gemcitabine for locally advanced pancreatic cancer. Int J Radiat Oncol Biol Phys. 2010;78:735–42. 20171803 10.1016/j.ijrobp.2009.08.046

[25] Cao D, et al. Opportunities and challenges in targeted therapy and immunotherapy for pancreatic cancer. Expert Rev Mol Med. 2021;23:e21. 34906271 10.1017/erm.2021.26

[26] Liu X, Li Z, Wang Y. Advances in targeted therapy and immunotherapy for pancreatic cancer. Adv Biol. 2021;5:1900236. 10.1002/adbi.201900236 33729700

[27] Collins FS, Varmus H. A new initiative on precision medicine. N Engl J Med. 2015;372:793–5. 25635347 10.1056/NEJMp1500523 PMC5101938

[28] Ashley EA. The precision medicine initiative: a new national effort. JAMA. 2015;313:2119–20. 25928209 10.1001/jama.2015.3595

[29] Ferrari R. Writing narrative style literature reviews. Med Writ. 2015;24:230–5.

[30] Gregory AT, Denniss AR. An introduction to writing narrative and systematic reviews—tasks, tips and traps for aspiring authors. Heart Lung Circ. 2018;27:893–8. 29857977 10.1016/j.hlc.2018.03.027

[31] Hamilton W, Walter FM, Rubin G, Neal RD. Improving early diagnosis of symptomatic cancer. Nat Rev Clin Oncol. 2016;13:740–9. 27458007 10.1038/nrclinonc.2016.109

[32] Vincent A, Herman J, Schulick R, Hruban RH, Goggins M. Pancreatic cancer. Lancet. 2011;378:607–20. 21620466 10.1016/S0140-6736(10)62307-0 PMC3062508

[33] Pannala R, Basu A, Petersen GM, Chari ST. New-onset diabetes: a potential clue to the early diagnosis of pancreatic cancer. Lancet Oncol. 2009;10:88–95. 19111249 10.1016/S1470-2045(08)70337-1 PMC2795483

[34] Yang J, et al. Early screening and diagnosis strategies of pancreatic cancer: a comprehensive review. Cancer Commun. 2021;41:1257–74. 10.1002/cac2.12204 PMC8696234 34331845

[35] Kleeff J, et al. Chronic pancreatitis. Nat Rev Dis Prim. 2017;3:1–18. 10.1038/nrdp.2017.60 28880010

[36] Makohon-Moore A, Iacobuzio-Donahue CA. Pancreatic cancer biology and genetics from an evolutionary perspective. Nat Rev Cancer. 2016;16:553–65. 27444064 10.1038/nrc.2016.66 PMC5739515

[37] Hayashi A, Hong J, Iacobuzio-Donahue CA. The pancreatic cancer genome revisited. Nat Rev Gastroenterol Hepatol. 2021;18:469–81. 34089011 10.1038/s41575-021-00463-z

[38] Linehan A, O’Reilly M, McDermott R, O’Kane GM. Targeting KRAS mutations in pancreatic cancer: opportunities for future strategies. Front Med. 2024;11:1369136. 10.3389/fmed.2024.1369136 PMC10991798 38576709

[39] Buscail L, Bournet B, Cordelier P. Role of oncogenic KRAS in the diagnosis, prognosis and treatment of pancreatic cancer. Nat Rev Gastroenterol Hepatol. 2020;17:153–68. 32005945 10.1038/s41575-019-0245-4

[40] Huang L, Guo Z, Wang F, Fu L. *KRAS* mutation: from undruggable to druggable in cancer. Signal Transduct Target Ther. 2021;6:386. 34776511 10.1038/s41392-021-00780-4 PMC8591115

[41] Yang X, Wu H. RAS signaling in carcinogenesis, cancer therapy and resistance mechanisms. J Hematol Oncol. 2024;17:108. 39522047 10.1186/s13045-024-01631-9 PMC11550559

[42] Dilly J, et al. Mechanisms of resistance to oncogenic KRAS inhibition in pancreatic cancer. Cancer Discov. 2024;14:2135–61. 38975874 10.1158/2159-8290.CD-24-0177 PMC11528210

[43] Waddell N, et al. Whole genomes redefine the mutational landscape of pancreatic cancer. Nature. 2015;518:495–501. 25719666 10.1038/nature14169 PMC4523082

[44] Olive K P. et al. Inhibition of Hedgehog signaling enhances delivery of chemotherapy in a mouse model of pancreatic cancer. Science. 2009;(80-. ). 324, 1457–1461. 10.1126/science.1171362 PMC2998180 19460966

[45] Provenzano PP, et al. Enzymatic targeting of the stroma ablates physical barriers to treatment of pancreatic ductal adenocarcinoma. Cancer Cell. 2012;21:418–29. 22439937 10.1016/j.ccr.2012.01.007 PMC3371414

[46] Herting CJ, Karpovsky I, Lesinski GB. The tumor microenvironment in pancreatic ductal adenocarcinoma: current perspectives and future directions. Cancer Metastasis Rev. 2021:1–15. 10.1007/s10555-021-09988-w. 10.1007/s10555-021-09988-w 34591240

[47] Sherman MH, Beatty GL. Tumor microenvironment in pancreatic cancer pathogenesis and therapeutic resistance. Annu Rev Pathol Mech Dis. 2023;18:123–48. 10.1146/annurev-pathmechdis-031621-024600 PMC9877114 36130070

[48] Zhang T, Ren Y, Yang P, Wang J, Zhou H. Cancer-associated fibroblasts in pancreatic ductal adenocarcinoma. Cell Death Dis. 2022;13:897. 36284087 10.1038/s41419-022-05351-1 PMC9596464

[49] Malik R, et al. Rigidity controls human desmoplastic matrix anisotropy to enable pancreatic cancer cell spread via extracellular signal-regulated kinase 2. Matrix Biol. 2019;81:50–69. 30412725 10.1016/j.matbio.2018.11.001 PMC6504628

[50] Ullman NA, Burchard PR, Dunne RF, Linehan DC. Immunologic strategies in pancreatic cancer: making cold tumors hot. J Clin Oncol. 2022;40:2789–805. 35839445 10.1200/JCO.21.02616 PMC9390820

[51] Vonderheide RH, Bear AS. Tumor-derived myeloid cell chemoattractants and T cell exclusion in pancreatic cancer. Front Immunol. 2020;11:605619. 33304355 10.3389/fimmu.2020.605619 PMC7693439

[52] Saadh MJ, et al. Targeting the pancreatic tumor microenvironment by plant-derived products and their nanoformulations. Med Oncol. 2024;41:201. 39001987 10.1007/s12032-024-02443-0

[53] Viale A, et al. Oncogene ablation-resistant pancreatic cancer cells depend on mitochondrial function. Nature. 2014;514:628–32. 25119024 10.1038/nature13611 PMC4376130

[54] Neesse A, Algül H, Tuveson DA, Gress TM. Stromal biology and therapy in pancreatic cancer: a changing paradigm. Gut. 2015;64:1476–84. 25994217 10.1136/gutjnl-2015-309304

[55] Li X, Thirumalai D. Cooperation among tumor cell subpopulations leads to intratumor heterogeneity. Biophys Rev Lett. 2020;15:99–119.

[56] Palma AM, Vudatha V, Peixoto ML, Madan E. Tumor heterogeneity: an oncogenic driver of PDAC progression and therapy resistance under stress conditions. Adv Cancer Res. 2023;159:203–49. 37268397 10.1016/bs.acr.2023.02.005

[57] Li X, Thirumalai D. Share, but unequally: a plausible mechanism for emergence and maintenance of intratumour heterogeneity. J R Soc Interface. 2019;16:20180820. 30958159 10.1098/rsif.2018.0820 PMC6364648

[58] Montalvo-Javé EE, et al. Pancreatic cancer: genetic conditions and epigenetic alterations. J Gastrointest Surg. 2023;27:1001–10. 36749558 10.1007/s11605-022-05553-0

[59] Conroy T, et al. FOLFIRINOX versus gemcitabine for metastatic pancreatic cancer. N Engl J Med. 2011;364:1817–25. 21561347 10.1056/NEJMoa1011923

[60] Milella M. FOLFIRINOX versus gemcitabine for metastatic pancreatic cancer. N Engl J Med. 2011:768–769. 10.1056/NEJMoa1011923.

[61] Suker M, et al. FOLFIRINOX for locally advanced pancreatic cancer: a systematic review and patient-level meta-analysis. Lancet Oncol. 2016;17:801–10. 27160474 10.1016/S1470-2045(16)00172-8 PMC5527756

[62] Von Hoff DD, et al. Increased survival in pancreatic cancer with nab-paclitaxel plus gemcitabine. N Engl J Med. 2013;369:1691–703. 24131140 10.1056/NEJMoa1304369 PMC4631139

[63] Zhang B, et al. The role of FOLFIRINOX in metastatic pancreatic cancer: a meta-analysis. World J Surg Oncol. 2021;19:182. 34154596 10.1186/s12957-021-02291-6 PMC8218408

[64] Tong H, Fan Z, Liu B, Lu T. The benefits of modified FOLFIRINOX for advanced pancreatic cancer and its induced adverse events: a systematic review and meta-analysis. Sci Rep. 2018;8:1–8. 29875415 10.1038/s41598-018-26811-9 PMC5989209

[65] Canon J, et al. The clinical KRAS (G12C) inhibitor AMG 510 drives anti-tumour immunity. Nature. 2019;575:217–23. 31666701 10.1038/s41586-019-1694-1

[66] Moore AR, Rosenberg SC, McCormick F, Malek S. RAS-targeted therapies: is the undruggable drugged? Nat Rev Drug Discov. 2020;19:533–52. 32528145 10.1038/s41573-020-0068-6 PMC7809886

[67] Kempf E, Rousseau B, Besse B, Paz-Ares L. KRAS oncogene in lung cancer: focus on molecularly driven clinical trials. Eur Respir Rev. 2016;25:71–6. 26929424 10.1183/16000617.0071-2015 PMC9487658

[68] Miyazaki S, et al. Targeting KRAS-mutant pancreatic cancer through simultaneous inhibition of KRAS, MEK, and JAK2. Mol Oncol. 2025. 10.1002/1878-0261.13751. 39400496 10.1002/1878-0261.13751 PMC11793007

[69] Contreras CT. et al. KRAS G12C-inhibitor-based combination therapies for pancreatic cancer: insights from drug screening. Mol Oncol. 10.1002/1878-0261.13725. 10.1002/1878-0261.13725 PMC11792994 39253995

[70] Strickler JH, et al. Sotorasib in KRAS p. G12C–mutated advanced pancreatic cancer. N Engl J Med. 2023;388:33–43. 36546651 10.1056/NEJMoa2208470 PMC10506456

[71] Hong DS, et al. *KRASG12C* inhibition with sotorasib in advanced solid tumors. N Engl J Med. 2020;383:1207–17. 32955176 10.1056/NEJMoa1917239 PMC7571518

[72] Hallin J, et al. The KRASG12C inhibitor MRTX849 provides insight toward therapeutic susceptibility of KRAS-mutant cancers in mouse models and patients. Cancer Discov. 2020;10:54–71. 31658955 10.1158/2159-8290.CD-19-1167 PMC6954325

[73] Le DT. et al. Mismatch repair deficiency predicts response of solid tumors to PD-1 blockade. Science. 2017 (80-. ). 357, 409–413. 10.1126/science.aan6733 PMC5576142 28596308

[74] Le DT, et al. PD-1 blockade in tumors with mismatch-repair deficiency. N Engl J Med. 2015;372:2509–20. 26028255 10.1056/NEJMoa1500596 PMC4481136

[75] O’Reilly EM, et al. Durvalumab with or without tremelimumab for patients with metastatic pancreatic ductal adenocarcinoma: a phase 2 randomized clinical trial. JAMA Oncol. 2019;5:1431–8. 31318392 10.1001/jamaoncol.2019.1588 PMC6647002

[76] Rojas LA, et al. Personalized RNA neoantigen vaccines stimulate T cells in pancreatic cancer. Nature. 2023;618:144–50. 37165196 10.1038/s41586-023-06063-y PMC10171177

[77] Ye X, Yu Y, Zheng X, Ma H. Clinical immunotherapy in pancreatic cancer. Cancer Immunol Immunother. 2024;73:64. 38430289 10.1007/s00262-024-03632-6 PMC10908626

[78] Fan J, et al. Current advances and outlooks in immunotherapy for pancreatic ductal adenocarcinoma. Mol Cancer. 2020;19:32. 32061257 10.1186/s12943-020-01151-3 PMC7023714

[79] Leidner R, et al. Neoantigen T-cell receptor gene therapy in pancreatic cancer. N Engl J Med. 2022;386:2112–9. 35648703 10.1056/NEJMoa2119662 PMC9531755

[80] Qi C, et al. CT041 CAR T cell therapy for Claudin18. 2-positive metastatic pancreatic cancer. J Hematol Oncol. 2023;16:102. 37689733 10.1186/s13045-023-01491-9 PMC10492318

[81] Zhong G, et al. Complete remission of advanced pancreatic cancer induced by claudin18. 2-targeted CAR-T cell therapy: a case report. Front Immunol. 2024;15:1325860. 38487523 10.3389/fimmu.2024.1325860 PMC10937427

[82] Rao AD, et al. Patient-reported outcomes of a multicenter phase 2 study investigating gemcitabine and stereotactic body radiation therapy in locally advanced pancreatic cancer. Pract Radiat Oncol. 2016;6:417–24. 27552809 10.1016/j.prro.2016.05.005 PMC5572652

[83] Arcelli A, et al. Higher biologically effective dose predicts survival in SBRT of pancreatic cancer: a multicentric analysis (PAULA-1). Anticancer Res. 2020;40:465–72. 31892602 10.21873/anticanres.13975

[84] Narayanan, G. Irreversible electroporation for treatment of liver cancer. Gastroenterol Hepatol (N. Y). 2011;7:313. PMC3127037 21857833

[85] Gajewska-Naryniecka A, et al. Irreversible electroporation in pancreatic cancer—an evolving experimental and clinical method. Int J Mol Sci. 2023;24:4381. 36901812 10.3390/ijms24054381 PMC10002122

[86] Moir J, White SA, French JJ, Littler P, Manas DM. Systematic review of irreversible electroporation in the treatment of advanced pancreatic cancer. Eur J Surg Oncol. 2014;40:1598–604. 25307210 10.1016/j.ejso.2014.08.480

[87] Rwigema J-CM, et al. Stereotactic body radiotherapy in the treatment of advanced adenocarcinoma of the pancreas. Am J Clin Oncol. 2011;34:63–9. 20308870 10.1097/COC.0b013e3181d270b4

[88] Thomson KR, et al. Investigation of the safety of irreversible electroporation in humans. J Vasc Interv Radiol. 2011;22:611–21. 21439847 10.1016/j.jvir.2010.12.014

[89] Lafranceschina S, et al. Systematic review of irreversible electroporation role in management of locally advanced pancreatic cancer. Cancers (Basel). 2019;11:1718. 31684186 10.3390/cancers11111718 PMC6896066

[90] Hezel AF, Kimmelman AC, Stanger BZ, Bardeesy N, DePinho RA. Genetics and biology of pancreatic ductal adenocarcinoma. Genes Dev. 2006;20:1218–49. 16702400 10.1101/gad.1415606

[91] Hruban RH, Goggins M, Parsons J, Kern SE. Progression model for pancreatic cancer. Clin Cancer Res. 2000;6:2969–72. 10955772

[92] Golan T, et al. Maintenance olaparib for germline BRCA-mutated metastatic pancreatic cancer. N Engl J Med. 2019;381:317–27. 31157963 10.1056/NEJMoa1903387 PMC6810605

[93] Luo G, et al. Pancreatic cancer: BRCA mutation and personalized treatment. Expert Rev Anticancer Ther. 2015;15:1223–31. 26402249 10.1586/14737140.2015.1086271

[94] Raphael KL, Willingham FF. Hereditary pancreatitis: current perspectives. Clin Exp Gastroenterol. 2016:197–207. 10.2147/CEG.S84358. 10.2147/CEG.S84358 PMC4968666 27555793

[95] Fang Y, et al. Genetic and molecular alterations in pancreatic cancer: implications for personalized medicine. Med Sci Monit. 2013;19:916. 24172537 10.12659/MSM.889636 PMC3818103

[96] Siciliano AC, et al. A 3D pancreatic cancer model with integrated optical sensors for noninvasive metabolism monitoring and drug screening. Adv Healthc Mater. 2024;13:2401138. 10.1002/adhm.202401138 38978424

[97] Wishart G, Gupta P, Schettino G, Nisbet A, Velliou E. 3D tissue models as tools for radiotherapy screening for pancreatic cancer. Br J Radiol. 2021;94:20201397. 33684308 10.1259/bjr.20201397 PMC8010544

[98] Tomás-Bort E, Kieler M, Sharma S, Candido JB, Loessner D. 3D approaches to model the tumor microenvironment of pancreatic cancer. Theranostics. 2020;10:5074. 32308769 10.7150/thno.42441 PMC7163433

[99] Cohen JD, et al. Detection and localization of surgically resectable cancers with a multi-analyte blood test. Science. 2018;359:926–30. 29348365 10.1126/science.aar3247 PMC6080308

[100] Thomas D, Radhakrishnan P. Tumor-stromal crosstalk in pancreatic cancer and tissue fibrosis. Mol Cancer. 2019;18:14. 30665410 10.1186/s12943-018-0927-5 PMC6341551

[101] Martinez-Bosch N, Vinaixa J, Navarro P. Immune evasion in pancreatic cancer: from mechanisms to therapy. Cancers (Basel). 2018;10:6. 29301364 10.3390/cancers10010006 PMC5789356

[102] Caronni N, et al. IL-1β+ macrophages fuel pathogenic inflammation in pancreatic cancer. Nature. 2023;623:415–22. 37914939 10.1038/s41586-023-06685-2

[103] Luo H, et al. Tumor-associated neutrophils upregulate Nectin2 expression, creating the immunosuppressive microenvironment in pancreatic ductal adenocarcinoma. J Exp Clin Cancer Res. 2024;43:258. 39261943 10.1186/s13046-024-03178-6 PMC11389261

[104] Bailey P, et al. Genomic analyses identify molecular subtypes of pancreatic cancer. Nature. 2016;531:47–52. 26909576 10.1038/nature16965

[105] Silva HM. mRNA technology in modern medicine: review and future prospects. Clin Mol Epidemiol. 2025;2:1–18.

[106] Sautès-Fridman C, et al. Tertiary lymphoid structures in cancers: prognostic value, regulation, and manipulation for therapeutic intervention. Front Immunol. 2016;7:407. 27752258 10.3389/fimmu.2016.00407 PMC5046074

[107] McGranahan N, et al. Allele-specific HLA loss and immune escape in lung cancer evolution. Cell. 2017;171:1259–71. 29107330 10.1016/j.cell.2017.10.001 PMC5720478

[108] Tao Z, et al. Impact of T cell characteristics on CAR-T cell therapy in hematological malignancies. Blood Cancer J. 2024;14:1–10. 39627220 10.1038/s41408-024-01193-6 PMC11615218

[109] Hou AJ, Chen LC, Chen YY. Navigating CAR-T cells through the solid-tumour microenvironment. Nat Rev Drug Discov. 2021;20:531–50. 33972771 10.1038/s41573-021-00189-2

[110] Wang AX, Ong XJ, D’Souza C, Neeson PJ, Zhu JJ. Combining chemotherapy with CAR-T cell therapy in treating solid tumors. Front Immunol. 2023;14:1140541. 36949946 10.3389/fimmu.2023.1140541 PMC10026332

[111] Qin VM, Haynes NM, D’Souza C, Neeson PJ, Zhu JJ. CAR-T plus radiotherapy: a promising combination for immunosuppressive tumors. Front Immunol. 2022;12:813832. 35095911 10.3389/fimmu.2021.813832 PMC8790144

[112] Grosser R, Cherkassky L, Chintala N, Adusumilli PS. Combination immunotherapy with CAR T cells and checkpoint blockade for the treatment of solid tumors. Cancer Cell. 2019;36:471–82. 31715131 10.1016/j.ccell.2019.09.006 PMC7171534

[113] Yamaguchi Y. et al. PD-L1 blockade restores CAR T cell activity through IFN-γ-regulation of CD163+ M2 macrophages. J Immunother Cancer. 2022;10. 10.1136/jitc-2021-004400 PMC9226933 35738799

[114] Fu R, et al. Combination therapy with oncolytic virus and T cells or mRNA vaccine amplifies antitumor effects. Signal Transduct Target Ther. 2024;9:118. 38702343 10.1038/s41392-024-01824-1 PMC11068743

[115] Gaertner J, Wolf J, Voltz R. Early palliative care for patients with metastatic cancer. Curr Opin Oncol. 2012;24:357–62. 22476189 10.1097/CCO.0b013e328352ea20

